# The zebrafish heart regenerates after cryoinjury-induced myocardial infarction

**DOI:** 10.1186/1471-213X-11-21

**Published:** 2011-04-07

**Authors:** Fabian Chablais, Julia Veit, Gregor Rainer, Anna Jaźwińska

**Affiliations:** 1 Department of Medicine, Unit of Anatomy, University of Fribourg, Fribourg, Switzerland; 2 Department of Medicine, Unit of Physiology, University of Fribourg, Fribourg, Switzerland; 3 Department of Biology, Unit of Zoology, University of Fribourg, Fribourg, Switzerland

## Abstract

**Background:**

In humans, myocardial infarction is characterized by irreversible loss of heart tissue, which becomes replaced with a fibrous scar. By contrast, teleost fish and urodele amphibians are capable of heart regeneration after a partial amputation. However, due to the lack of a suitable infarct model, it is not known how these animals respond to myocardial infarction.

**Results:**

Here, we have established a heart infarct model in zebrafish using cryoinjury. In contrast to the common method of partial resection, cryoinjury results in massive cell death within 20% of the ventricular wall, similar to that observed in mammalian infarcts. As in mammals, the initial stages of the injury response include thrombosis, accumulation of fibroblasts and collagen deposition. However, at later stages, cardiac cells can enter the cell cycle and invade the infarct area in zebrafish. In the subsequent two months, fibrotic scar tissue is progressively eliminated by cell apoptosis and becomes replaced with a new myocardium, resulting in scarless regeneration. We show that tissue remodeling at the myocardial-infarct border zone is associated with accumulation of Vimentin-positive fibroblasts and with expression of an extracellular matrix protein Tenascin-C. Electrocardiogram analysis demonstrated that the reconstitution of the cardiac muscle leads to the restoration of the heart function.

**Conclusions:**

We developed a new cryoinjury model to induce myocardial infarction in zebrafish. Although the initial stages following cryoinjury resemble typical healing in mammals, the zebrafish heart is capable of structural and functional regeneration. Understanding the key healing processes after myocardial infarction in zebrafish may result in identification of the barriers to efficient cardiac regeneration in mammals.

## Background

Cardiovascular diseases in humans frequently manifest in acute myocardial infarction, commonly known as a heart attack, which causes muscle cell death due to lack of oxygenation [1,2]. In surviving patients, the dead myocardium is eventually replaced by a collagen-rich scar, which leads to pathologies, including further infarctions. In addition, remaining cardiomyocytes, the major cardiac structural cells, undergo cell enlargement to compensate for the loss of tissue. This results in incomplete restoration of the heart function. Most evidence to date indicates that cardiomyocyte proliferation, which represents a more effective way to replace missing tissue, does not significantly contribute to the mammalian injury response [3,4]. Thus, inability of adult cardiomyocytes to re-enter the cell cycle is considered as the main limitation of the poor cardiac regenerative potential in mammals [5].

Non-mammalian vertebrates capable of heart regeneration, such as urodele amphibians and teleost fish, reconstitute the myocardium with only moderate scarring in newts, or with little or no scarring in zebrafish [6-8]. In contrast to mammals, adult newt and zebrafish cardiomyocytes can dedifferentiate and re-enter the cell cycle [9-13]. However, the molecular and cellular mechanisms underlying heart regeneration in these model organisms are still very poorly understood. Comparative analysis between animals with different capacities to regenerate their heart will advance our understanding and allow the development of strategies to limit scarring and enhance myocyte restoration after heart injuries in humans.

Currently, the research on heart regeneration in newts and zebrafish is based on mechanically-induced injuries [14-16]. Zebrafish was shown to survive resection of up to 20% of the ventricle [15,17]. Shortly after amputation, a fibrotic clot fills up the wound site and the injured area contracts. BrdU-cell-proliferation assay demonstrated that starting from day 7, a subset of cardiomyocytes synthesizes DNA, and cardiac muscle invades the injury site. In one to two months, the regeneration process is completed, as seen by replacement of fibrotic tissue with new myocardium.

The traditional approach to surgically remove the ventricular apex is not very representative for infarcts encountered in mammalian systems, which is associated with massive cell death within the myocardial wall [18]. Cell death triggers rapid activation of the immune system, generates free radicals, and induces a protease-rich environment leading to extensive degradation of the dead matrix [19,20]. These conditions have been shown to affect the efficiency of tissue regeneration [19,20]. However, to date, no study has been performed to elucidate zebrafish heart regeneration after a heart infarct. Several models of myocardial infarction have been proposed in various mammalian organisms, including permanent ligation of the coronary artery or cryoinjury [1,21,22]. In this study, we adapted the cryoinjury method to induce a heart infarct in zebrafish, because of the coronary vasculature is not accessible to manipulations [23]. We show that the scar tissue forms only transiently and it is replaced with a new myocardium within two months. Studies in mammals demonstrated a critical role of fibroblasts and the extracellular matrix for the reparative response following myocardial infarction [24,25]. Therefore, a particular focus of this study is toward characterization of non-cardiac cells during heart regeneration in zebrafish.

## Methods

### Animal Procedures

The following zebrafish strains were used in this study: wild-type AB (Oregon) strain, Ekkwil (EK) strain, transgenic strain cmlc2::DsRed2-nuc [26] to visualize cardiomyocytes nuclei, and transgenic strain cmlc2::EGFP [27] to analyze the injured area on the whole hearts. Fish aged 6-18 months were anesthetized in 0.1% tricaine (Sigma Aldrich) and placed ventral side up in a damp sponge. A small incision was made through the chest with iridectomy scissors to access the heart. The ventricular wall was directly frozen by applying for 23-25 seconds a stainless steel cryoprobe precooled in liquid nitrogen. The tip of the cryoprobe was 6 mm long with a diameter of 0.8 mm, the handle of the cryoprobe was 4 cm long with a diameter of 8 mm and was covered with a plastic surface. To stop the freezing of the heart, fish water at room temperature was dropped on the tip of the cryoprobe. For heart resection surgeries, ventricular muscle was removed at the apex with iridectomy scissors as previously described [15]. Animals were allowed to regenerate for various times at 26.5°C. Experimental research on animals has been approved by the cantonal veterinary office of Fribourg.

### Histological techniques

Acid Fuchsin Orange-G (AFOG) staining was performed as described previously [15]. For Hematoxylin and Eosin staining, hearts were fixed in 2% Formalin overnight at 4°C, dehydrated and embedded in paraffin blocks. Sections were cut at the thickness of 6 μm, rehydrated and stained with Mayer's Haemalum for 12 minutes. The nuclear staining was differentiated for 5 seconds in 0.37% HCl prepared in 70% ethanol, and the slides were washed in tap water for 10 minutes. The staining of proteins was obtained by incubation for 10 minutes in 0.1% Eosin Y solution in water with a drop of acidic acid, followed by a rapid wash in water. The sections were dehydrated in a water/ethanol series, cleared in xylol, and mounted in Entelan medium (Merck). For morphometric analysis, all consecutive section of 6 hearts per time point were photographed using a microscope and Leica DFC480 camera. The infarct region and the intact myocardium were demarcated, and the areas were measured using ImageJ software. The percentage of the infarct size relative to the entire ventricle was calculated.

### Immunohistochemistry

The primary antibodies used in this study were: rabbit anti-MEF-2 at 1:50 (Santa Cruz Biotechnology); mouse anti-Tropomyosin at 1:100 (developed by Jim Jung-Ching Lin and obtained from the Developmental Studies Hybridoma Bank, The University of Iowa); rabbit anti-Tenascin-C at 1:500 (USBiological); mouse anti-Vimentin at 1:70 (developed by Arturo Alvarez-Buylla and obtained from the Developmental Studies Hybridoma Bank, The University of Iowa); rabbit anti-alpha Smooth Muscle Actin at 1:200 (GeneTex), rabbit anti-MCM5 at 1:5000 (kindly provided by Soojin Ryu). The secondary antibodies used in this study at a concentration of 1:500 were: goat anti-rabbit Alexa Fluor 488, goat anti-mouse Alexa Fluor 488 (Molecular Probes); goat anti-rabbit Cy3 conjugated, goat anti-rabbit Cy5, goat anti-Mouse DyLight 549 or 649 conjugated (Jackson ImmunoResearch). DAPI (Roche) was used at a concentration 1:1000.

The hearts were fixed overnight at 4°C in 2% formalin, washed several times in PBS, equilibrated in 30% sucrose, cryosectioned at the thickness of 16 μm and immunostained as previously described [28]. The specimens were analyzed with confocal microscopy (Leica TCS SP5). For the quantification of proliferating cardiomyocytes, MCM5/DsRed2 double positive nuclei were normalized as the percentage of the total number of DsRed2-positive nuclei using the software ImageJ 1.42 h.

### Terminal deoxynucleotidyl transferase dig-dUTP nick end-labeling (TUNEL)

For TUNEL reactions, the cryosections were post-fixed for 10 minutes in 1% formalin, washed twice 5 minutes in PBS and pretreated in precooled ethanol:acetic acid 2:1 for 5 minutes at -20°C. After washing in PBS, DNA breaks were elongated with Terminal Transferase (Roche) and Digoxigenin-dUTP solution (Roche) as described [29]. The reaction was stopped by incubation in 300 mM NaCl, 30 mM sodium citrate for 10 min, followed by washing in PBS. The staining with anti-digoxigenin fluorescein conjugated was performed according to manufacturer protocol (Roche). After a wash in PBS, the sections were used for immunohistochemistry as described above.

### ECG recordings and acquisition

All the ECGs were recorded in triplicates before injury and at the subsequent time points after surgery. To reduce the biological variations, the same six animals were used for recordings before injury and at the subsequent time points after surgery. The fish were anesthetized by immersion in 0.1% tricaine solution for 90 seconds. Anesthetized zebrafish were placed ventral side up in a sponge. Two 29-gauge stainless steel micro-electrodes (AD Instrument, Colorado Springs, CO) were positioned with a micromanipulator as described [30]. Each ECG was recorded for 45 seconds and then the fish were allowed to recover in water free of tricaine. ECGs acquisitions were made with a Tektronix 5A22N differential amplifier. Signals were sampled at 6.1 kHz and band-pass filtered between 1 and 4000 Hz with a TDT System 3 RZ5 Neurophysiology Workstation (Tucker-Davis Technologies). Digital Signals were then low-pass filtered below 30 Hz and the RR and QT interval were automatically extracted by custom made software written in MATLAB R2007b (The Mathworks Inc.). Each trace was visually examined before accepting the automatic calculations. QT intervals were normalized to the heart rates using the standard Bazett formula: QTc = QT/(RR^1/2^)

## Results

### Massive cell apoptosis distinguishes cryoinjury from ventricular resection

**Figure 1:**
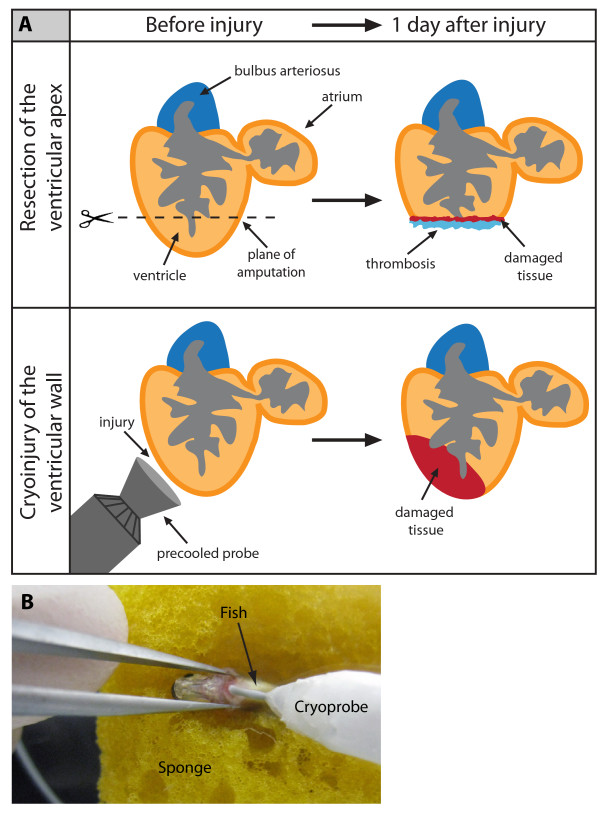
**Cyroinjury compared to resection**. **(A) **After amputation of the ventricular apex (upper panel), the damaged myocardium is restricted to the narrow layer of cells above the amputation plane. A blood clot fills up the missing tissue below the plane of amputation. After cryoinjury (lower panel), a large portion of the damaged apoptotic myocardium (red) remains integrated with the functioning organ. **(B) **A photograph of the cryoinjury procedure. A small incision of the zebrafish chest is opened with help of forceps. The cryoprobe is placed in contact with the ventricle.

**Figure 2:**
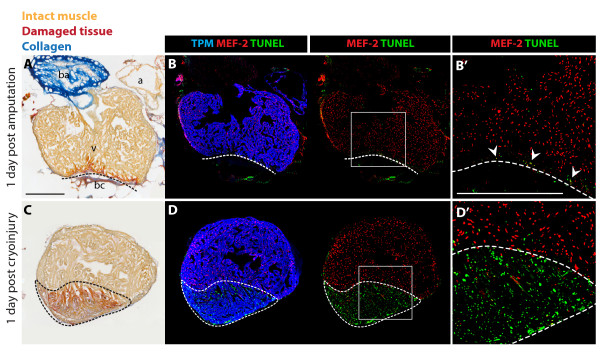
**Massive cell apoptosis distinguishes cryoinjury from ventricular resection**. **(A and C) **AFOG (Acid Fuchsin Orange-G) staining labels healthy muscle cells (light orange), damaged cells with plasma (dark orange), and collagen (blue). **(B and D) **Immunostaining with Tropomyosin (TPM) for cardiac muscle (blue), and nuclear MEF-2 as a marker for healthy cardiomyocytes (red). Apoptotic cells are detected by the TUNEL assay (green). **(B' and D') **Higher magnifications of the framed area in the left panels. **(A-B) **Adjacent longitudinal sections of the heart one day after ventricular resection. **(A) **The ventricle (v) comprises a narrow stripe of damaged cardiomyocytes (dark orange) just above the amputation plane (dashed line). Underneath the amputation plane a blood clot (bc) seals the wound; a, atrium; ba, bulbus arteriosus. **(B-B') **A narrow layer of damaged TPM-positive myocardium above the amputation plane (dashed line) display enhanced apoptosis and reduced MEF-2 expression (arrowheads). **(C-D) **Adjacent cross sections of the heart one day after cryoinjury. **(C) **The ventricle encompasses a large disk-shaped damage (encircled with dashed line). **(D-D') **This region contains abundant apoptotic TPM-positive cardiomyocytes that downregulate MEF-2 expression. Scale bars in **(A-B')**, 300 μm.

To investigate the regenerative capacity of the zebrafish heart in response to myocardial infarction, we developed a new injury method by freezing-thawing tissue, referred to as cryoinjury. In contrast to the previously used technique of ventricular resection [15], our approach aimed to mirror mammalian infarcts associated with massive cell death and inflammation (Figure 1). The histological AFOG (Acid Fuchsin Orange-G) staining of heart sections at 1 day post surgery revealed a disc-shaped infarct after cryoinjury, encompassing a large part of the ventricular wall, whereas only a thin layer of damaged tissue above the plane of amputation was observed after resection (Figure 2A, C). In contrast to the resection model, cryoinjury resulted in the extensive death of cardiomyocytes, as detected by the TUNEL cell apoptosis assay (Figure 2B-B', D-D'). Such damage is reminiscent of a heart infarct in mammals [20]. Another advantage of our new approach is a very low total mortality of animals during the first day after the procedure (5%, n = 200), when compared to the mortality caused by ventricular resection (25%, n = 20).

### Complete regeneration after myocardial infarction

**Figure 3:**
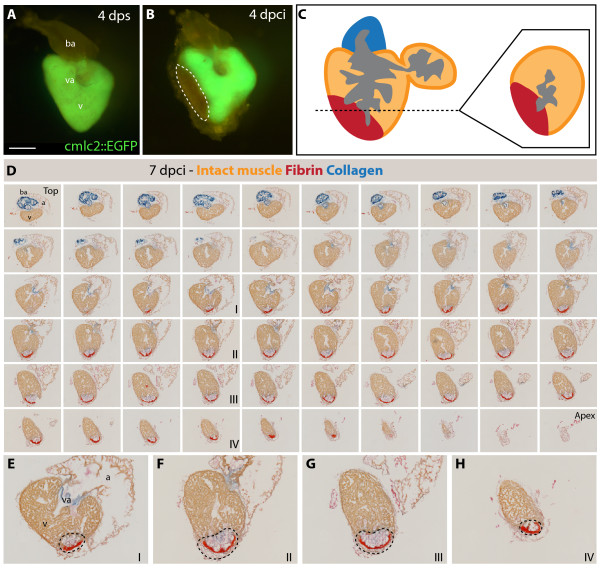
**Analysis of the infarct size after cryoinjury**. **(A-B) **Whole hearts of transgenic fish expressing GFP under control of cardiac specific promoter *cmlc-2*. **(A) **Uninjured ventricle at 4 days post sham (dps); v, ventricle; va, valve; ba, bulbus arteriosus; scale bar 300 μm. **(B) **At 4 days post cryoinjury (dpci), a portion of the ventricular wall is devoid of GFP expression, indicating the damaged myocardium (encircled by dashed line). **(C) **Schematic drawing demonstrating the plane of sectioning of the heart, which we applied in all our analysis. A typical cross-section is shown at the right side of the panel. **(D) **7 dpci, a consecutive series of cross-sections of one heart from the top of the ventricle (left upper corner) to the ventricular apex (right bottom corner) labeled to AFOG staining to visualize the healthy myocardium in orange, fibrin in red and collagen in blue. **(E-H) **Higher magnification of selected images shown in **(D)**. The post-infarct zone (black dashed line) expands from the apex to approximately a half-length of the ventricular wall. In our morphometric analysis, the measurement of the infarct volume was taken from all the sections of six hearts at different time points after injury. v, ventricle; va, valve; ba, bulbus arteriosus; a, atrium.

**Figure 4:**
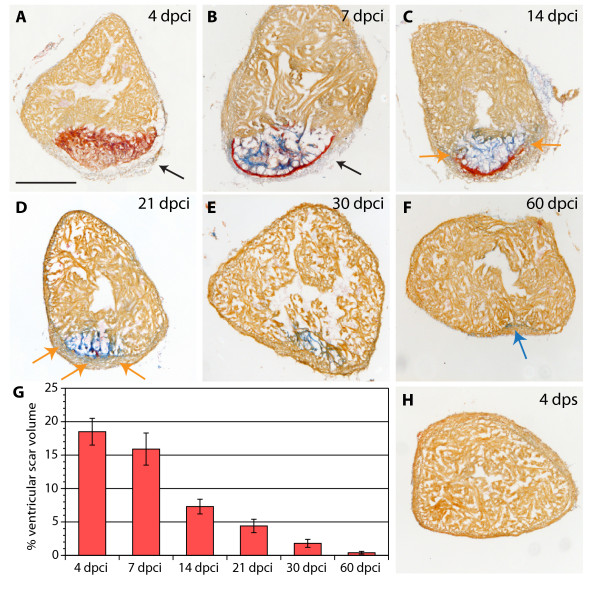
**Scar formation and resorption during healing of myocardial infarction after cryoinjury**. **(A-F and H) **Heart sections stained with AFOG representing the subsequent stages after cryoinjury. **(A) **At 4 dpci (days post cryoinjury), the damaged myocardium (dark orange) becomes surrounded by non-muscle cells (light gray, denoted by arrow). **(B) **At 7 dpci, a fibrin layer (red) forms along the inner side of the wound margin that consists of non-myocytes (arrow). The central part of the post-infarct consists of a loose collagen network (blue). **(C) **At 14 dpci, the edges of the fibrin layer (red) resolve and are replaced by new myocytes (orange arrow). The central part of the post-infarct contains abundant collagen fibers (blue). **(D) **At 21 dpci, a wall of cardiac tissue surrounds the entire infarct (orange arrows). Fibrin (red) is markedly reduced, while the collagen fibers (blue) persist. **(E) **At 30 dpci, no fibrin is visible. The collagen-containing area has markedly decreased. **(F) **At 60 dpci, the infarct scar is nearly completely resolved. Only occasionally, a few collagen fibers are detected (blue arrow). **(G) **A change of the scar size relative to the entire ventricle at different stages after injury. For measurements, all cross sections of six hearts per time point were analyzed. **(H) **In control, at 4 dps (days post sham), no fibrin or collagen fibers are present. Scale bar in **(A)**, 300 μm.

To determine the position of the infarct after our cryoinjury procedure, we used transgenic fish expressing GFP under the control of cardiac-specific *cmlc-2 *promoter. At 4 dpci (days post cryoinjury), the absence of GFP fluorescent signal in the dissected hearts demarcated the infarct zone along the apical-lateral ventricular wall (Figure 3A-B). To determine the extent of heart regeneration after cryoinjury, we performed histological AFOG-staining of all consecutive cross-sections of the heart (Figure 3C-H and Additional file 1). The infarct size was measured relative to the volume of the ventricle at different time points after the procedure (Figure 4G). The morphometric analysis of hearts at 4 dpci revealed that 18.5% ± 2.0 (n = 6) of the ventricular wall suffered from damage. A low SEM value (standard error of the mean) demonstrates the consistency of our method. A similar wound size was observed at 7 dpci (15.9% ± 2.4). At 14 dpci, the post-infarct area decreased by approximately 50% (7.3% ± 1.1), and further progressive reduction was observed at 21 dpci (4.1% ± 1.0), 30 dpci (1.8% ± 0.6) and finally 60 dpci (0.4%% ± 0.3), when the injured area was nearly absent. Thus, the infarct was completely replaced with the new myocardium within two months.

Next, we carefully characterized the histology of the post-infarct using AFOG and H&E staining. Cell death is known to invoke the recruitment of the inflammatory cells that remove the necrotic cell debris by phagocytosis [2,20]. At 4 dpci, the proinflammatory environment was associated with a deposition of fibrin and initiation of the reparative processes (Figure 4A). At 7 dpci, the fibrin-based provisional matrix was lysed, and most of the dead cells and debris were cleaned. At this time, two conspicuous scar structures appeared: a layer of fibrin that surrounded the outer border of the post-infarct, and a network of collagen in the interior of the wound (Figure 4B). In addition, mesenchymal cells accumulated around the fibrin-based matrix and the infarct was strongly infiltrated by blood cell (Additional file 2B). These histological findings are reminiscent of the fibrotic tissue built after mammalian infarcts [25,31].

While the deposition of collagen was further enriched at 14 dpci, the fibrin layer started to resolve at the boundaries abutting the healthy myocardium (Figure 4C, Additional file 2C and Additional file 3A-D). From this area, protrusions of cardiac tissue invaded the post-infarct. At 21 dpci, a new wall of compact myocardium surrounded the post-infarct border, replacing the fibrin layer (Figure 4D). At 30 dpci, fibrin decreased below detectable levels, only some collagen fibers persisted (Figure 4E and Additional file 3E-H). Residual collagen was cleared at 60 dpci, and the post-infarct zone was often undistinguishable from the uninjured myocardium (Figure 4F and 4H). This demonstrates that the extensive collagenous scar is fully resorbed and replaced by newly regenerated myocardium after two months post infarction of the apical-lateral ventricular wall.

### Enhanced cell apoptosis in the post-infarct and cardiomyocyte proliferation during heart regeneration

**Figure 5:**
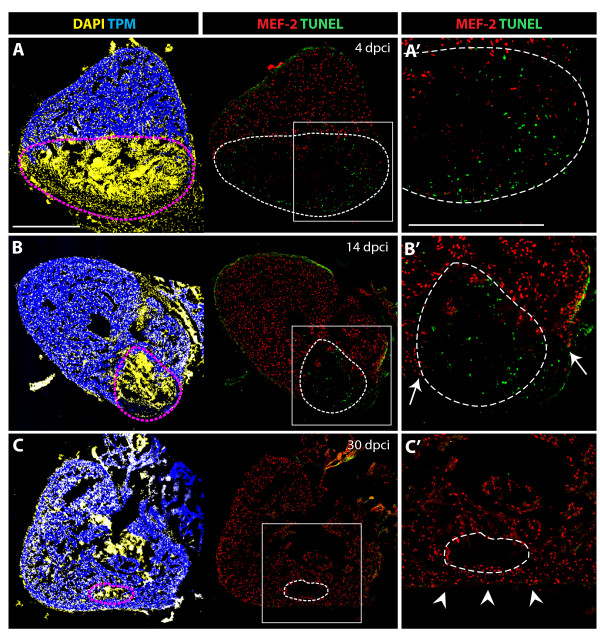
**Cell apoptosis during the replacement of the scar with a new myocardium**. **(A-C) **Confocal images of quadruple-stained cross-sections of hearts at different time points after cryoinjury. Cardiomyocytes are labeled by the presence of TPM (blue) and MEF-2 (red). All the nuclei are visualized by DAPI (yellow) and the apoptotic cells are detected by the TUNEL assay (green). Dashed lines encircle the scar. **(A', B' and C') **Higher magnifications of framed area shown in left panels. **(A-A') **At 4 dpci, a large infarct area is devoid of cardiomyocytes, and it contains abundant apoptotic cells. **(B-B') **At 14 dpci, numerous non-myocytes located in the infarct zone undergo apoptosis. Arrows indicate new myocardium invading the post-infarct. **(C-C') **At 30 dpci, cell apoptosis is no longer detected in the remaining post-infarct region. Cardiomyocytes have replaced a large portion of the wound, and only a small area lacks cardiomyocytes. Arrowheads denote a new myocardial wall. Scale bars in **(A-A') **represent 300 μm.

The replacement of the scar with new cardiac tissue requires removal of infarct cells and invasion of new cardiomyocytes. To assess the contribution of cell apoptosis in this process, we performed the TUNEL assay at different time points after injury (Figure 5). No TUNEL-positive cells were detected in the healthy myocardium, which was labeled by Tropomyosin/MEF-2 staining. In contrast, the post-infarct zone contained numerous apoptotic cells at 4 and 14 dpci (Figure 5A-A' and 5B-B'). No significant apoptosis was detected at 30 dpci, when fibrotic tissue was largely replaced by cardiomyocytes (Figure 5C-C'). This indicates that cell apoptosis is associated with the elimination of non-cardiac tissue during remodeling of the post-infarct.

**Figure 6:**
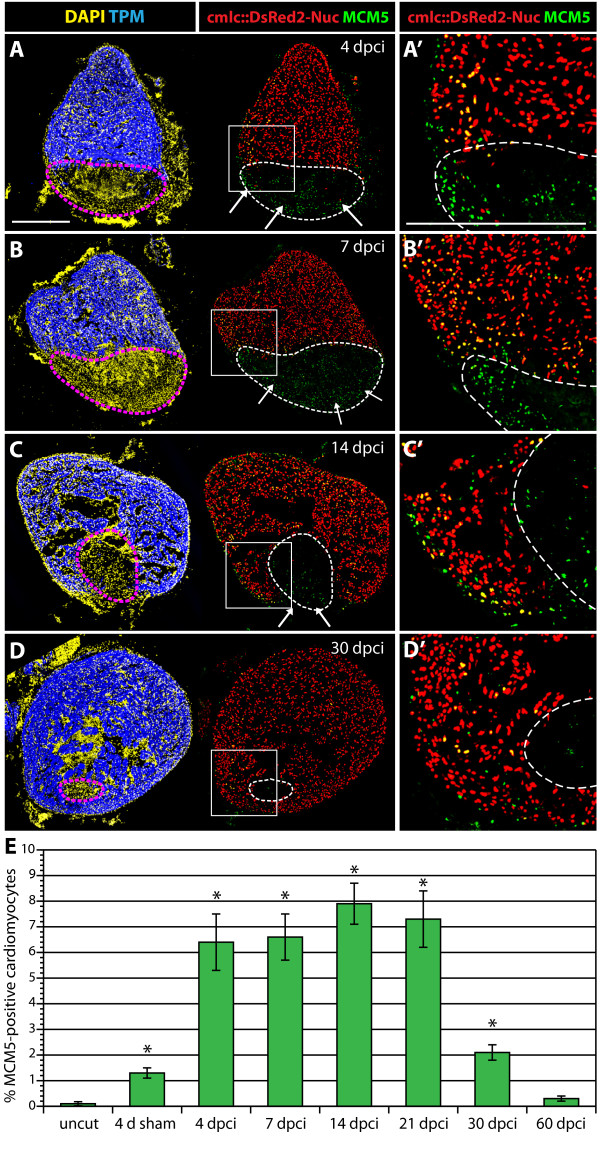
**Enhanced cardiomyocyte proliferation during heart regeneration**. **(A-D) **Confocal images of the heart from transgenic fish expressing nuclear DsRed2Nuc under the control of *cmlc-2 *cardiomyocyte-specific promoter. Immunostaining for Tropomyosin (blue) labels the myocardium, MCM5 (green) detects mitotic cells and DAPI (yellow in the left panels) marks all the nuclei. Proliferating cardiomyocytes are identified by the co-expression of DsRed2-Nuc and MCM5. Dashed line denotes the post-infarct. **(A', B', C' and D') **Higher magnifications of the framed area in the left panels. At 4 dpci **(A-A')**, 7 dpci **(B-B')**, 14 dpci **(C-C') **and 30 dpci **(D-D')**, many proliferating cardiomyocytes are located in the vicinity of the post-infarct. At 4, 7 and 14 dpci, a layer of proliferating non-myocytes surrounds the periphery of the scar (arrows in **A, B and C**). Bars in **(A and A')**, 300 μm. **(E) **Ratios of MCM5/DsRed2-Nuc-positive nuclei relative to DsRed2-Nuc nuclei. (n = 10, 2 representative sections of 5 hearts, *P < 0.01 compared to uncut).

For cell proliferation analysis, we needed to distinguish between cardiac and non-cardiac nuclei. Therefore, we used a transgenic strain of zebrafish that expresses nuclear DsRed2 under the control of *cardiac myosin light chain2 *promoter (*cmlc2::DsRed2-nuc*) [26]. To assess cell proliferation, we assayed cell cycle entry with the antibody against MCM5 (DNA replication licensing factor; Figure 6). In hearts of uninjured fish, the number of MCM5-positive cardiomyocytes was low (0.1% ± 0.02). Surprisingly, at four days after a sham operation, we detected a 10-fold increase (1.2% ± 0.2) in the number of MCM5/DsRed-positive cells. The majority of these cell-cycling cardiomyocytes were located in the vicinity of the epicardium (Additional file 4). This suggests that a sham operation caused a superficial disturbance and inflammation of the epicardium, which is sufficient to trigger cardiomyocyte proliferation.

In comparison to either uninjured or sham-operated fish, the animals that were subjected to cryoinjury displayed a noticeable enhancement of cardiomyocyte proliferation. As early as at 4 dpci, the proportion of proliferating cardiomyocytes increased to 6.4% ± 1.1 (Figure 6A-A' and Figure 6E). Similar values, ranging from 6 to 8%, were detected at subsequent time points of 7, 14 and 21 dpci (Figure 6B-B', C-C' and Figure 6E). This finding is consistent with a stable rate of regeneration detected by our morphometric analysis of the post-infarct (Figure 4G). At 30 dpci, the number of proliferating cardiomyocytes decreased to 2.1% ± 0.3 (Figure 6D-D' and Figure 6E). At 60 dpci, no enhanced cardiomyocyte proliferation was observed, indicating termination of heart regeneration (Figure 6E). We conclude that reconstitution of the new myocardium is associated with mitotic activation of cardiac cells.

### Vimentin and Tenascin-C expression during post-infarct remodeling

**Figure 7:**
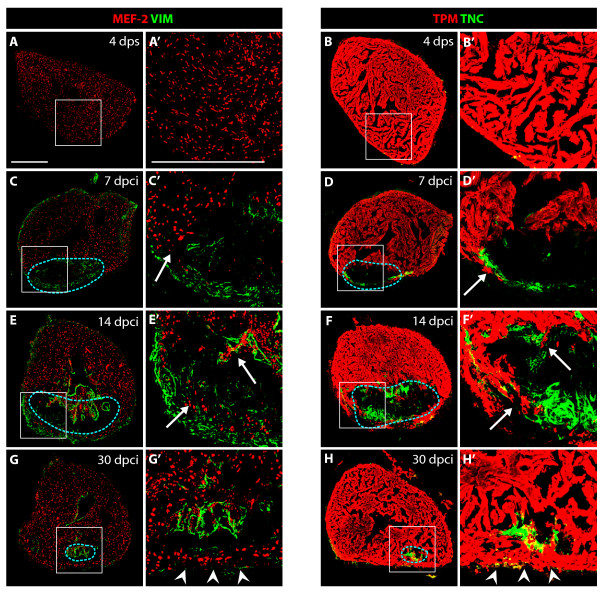
**VIM-positive fibroblasts and TNC localize at the border zone between the myocardium and fibrotic tissue**. **(A, C, E and G) **Heart sections immunostained for a cardiac nuclear marker MEF-2 (red) and an intermediate filament marker Vimentin (VIM, green). **(B, D, F and H) **Heart sections immunostained for a cardiac sarcomeric marker TPM (red) and an extracellular de-adhesive protein Tenascin-C (TNC, green). **(A', B', C', D', E', F', G' and H') **Higher magnifications of the framed area in the left panels. **(A-A' and B-B') **In control, at 4 days after sham operation, no significant expression of VIM and TNC can be detected in the ventricle. **(C-C' and D-D') **At 7 dpci, the scar margin and the interface between the myocardium and post-infarct are highlighted by VIM- and TNC-expressing fibroblasts. **(E-E' and F- F') **At 14 dpci, protrusions of cardiomyocytes expand along VIM/TNC-expressing cells (arrows). **(G-G' and H-H') **At 30 dpci, the scar tissue is largely replaced by cardiomyocytes. A new compact myocardial wall (arrowheads) surrounds residual VIM and TNC. Dashed line encircle the post-infarct. Bars in **(A-A')**, 300 μm.

Tissue replacement requires that the new cells rearrange their initial position and move into the adjacent areas. To identify spatiotemporal cues that may direct the migration of myocytes, we performed an antibody screen for molecules expressed at the infarct-myocardial boundary. Among the tested candidates, we found two such molecules: intermediate filament Vimentin (VIM) and extracellular de-adhesive protein Tenascin-C (TNC). VIM and TNC are known to participate in a number of critical cellular processes, such as adhesion, migration, and cell signaling in mammals and zebrafish [32-35]. In the vertebrate heart, anti-vimentin antibodies react with the abundant intermediate filaments of fibroblasts and some endothelial cells under mechanical stress [36]. TNC is upregulated by cardiac fibroblast during myocardial tissue remodeling under pathological conditions [37]. In adult zebrafish, both proteins were not detected in myocardium, epicardium or blood vessel cells of uninjured hearts, except for VIM at the base of valve leaflets and the outflow tract (Figure 7A-A', B-B', Additional file 5 and data not shown). During myocardial regeneration, at 7, 14 and 30 dpci, both markers were expressed around the injury site (Figure 7C-H'). The most conspicuous accumulation of TNC was at the border zone between the intact myocardium and the injury site. Since the edge of the residual myocardium is the most active site of cardiomyocyte migration, this characteristic localization suggests the particular role of TNC during the expansion of new myocardium. In contrast, the VIM-positive connective tissue contributed to the wall of the post-infarct at 7 and 14 dpci. These cells also expressed alpha smooth muscle actin (α-SMA), a marker of migratory, proliferative fibroblasts (Additional file 6) [38]. The rim of VIM- and α-SMA-positive cells may constitute a structural support-framework for the damaged ventricle, guiding restoration of cardiac architecture, while TNC counter-adhesive domains may facilitate cell migration at the edges of the myocardium.

### Functional regeneration of the heart

**Figure 8:**
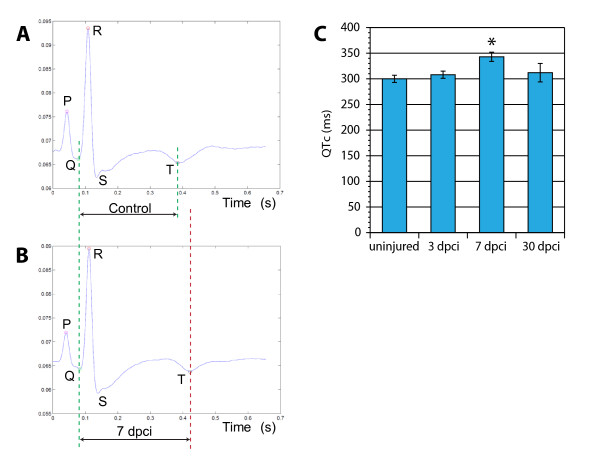
**Electrocardiogram (ECG) analysis following cryoinjury revealed a prolonged duration of the ventricular action potential in scar-containing hearts**. An average ECG of uninjured animals **(A) **and hearts at 7 dpci **(B) **demonstrates a similar pattern of waves except the prolonged time between the Q and T peaks. **(C) **Average duration of the QTc-intervals at different time points after cryoinjury (n = 6 fish, *P < 0.01).

Accumulation of fibrotic tissue stiffens the ventricles and impedes both contraction and relaxation. To assess the functional consequences of the cryoinjury, we recorded the heart conduction using electrocardiograms (ECGs). The ECG recordings of zebrafish were similar to a typical mammalian ECG with the characteristic peaks (Figure 8A and 8B). Specifically, an atrial excitation (P wave) is followed by a ventricular contraction (QRS wave), and repolarization (T wave). Thus, despite having a two-chambered heart, the zebrafish heart conduction pattern reflected that of humans [30]. Clinical studies have correlated persistent prolonged ventricular repolarization, displayed as QT intervals, with a risk of sudden death of patients after myocardial infarction [39,40]. To examine whether similar effects are caused by infarction in zebrafish, we determined QTc values of the same animals before and after cryoinjury. We found no significant difference between the QTc values before surgery and at 3 dpci. However, at 7 dpci, corresponding to the time of scar formation, the QTc values were markedly prolonged (Figure 8C). We concluded that the extensive fibrosis obstructs the spread of electrical stimuli by producing an insulating layer of collagen, resulting in elecrophysiological effects. Interestingly, at 30 dpci, the hearts with diminished scar displayed QTc values comparable to the control (Figure 8C). The regenerated hearts recovered their original electrical properties, indicating that the new cardiomyocyes were functionally integrated with the pre-existing myocardium.

## Discussion and Conclusions

Teleost fish, urodele amphibians and mammals represent groups of vertebrates with different cardiac regenerative potential [8,18,41]. Studies aimed at identifying these differences are difficult to interpret because the nature of injuries applied varies. Here, we developed a new standardized protocol of cryoinjury in zebrafish, which replicates the experimental procedure that has been described in mice, rats, rabbits and pigs [22]. We showed that in comparison to ventricular resection, cryoinjury results in a much more extensive cell death within the ventricular wall. We could show that zebrafish heart is capable of complete myocardial regeneration after infarction of up to 20% of the ventricle. Moreover, impaired heart function, which was characterized by prolonged ventricular action potential (QTc), becomes normalized within a month after cryoinjury. Thus, the zebrafish represents a valuable vertebrate model to study the response to myocardial infarction.

The cellular and molecular events during heart regeneration in zebrafish can be divided in three overlapping phases: First, a resolution of inflammation takes place, in which the infarct is cleared from dead cells by macrophages. Subsequently, the reparative phase begins with the formation of the fibrin layer that seals the wound, and accumulation of fibroblasts that produce a collagen scaffold. Finally, the regenerative phase follows, during which a new myocardium invades the infarct, while the fibrin-collagen based matrix undergoes progressive degradation. During this process, ventricular cardiomyocytes enter the cell cycle, which reveals their proliferative capacity. This finding is consistent with previous studies demonstrating the origin of the new myocardium from differentiated cardiomyocytes during heart regeneration after ventricular resection [10,11]. Although the initial two phases are also characteristic for healing of mammalian infarcts, the third one is unique to zebrafish. Remarkably, the collagen matrix, instead of becoming a mature scar, is replaced by new invading myocytes. Thus, the zebrafish heart can switch from the deposition to the degradation of fibrotic tissue. This supports the idea that regeneration can be considered as a process dependent on a concerted interplay between cardiomyocytes and nonmyocardial cells of the post-infarct [18].

Fibroblasts have been underestimated in the previous studies of zebrafish heart regeneration. In mammals, they are known to play a central role in the regulation of ECM production and degradation, cell migration and proliferation after myocardial infarction [24,31,38]. Therefore, a particular focus of this study is to characterize non-cardiac cells emerging at the infarct zone in zebrafish. We demonstrated enhanced fibroblast proliferation along the wall of the infarct. It is likely that enhanced fibroblast proliferation at the injury site compensates biomechanical deformation of the infracted ventricle, which requires a transient scar to withstand the blood pressure. A reduction of the ventricular wall stress, due to contractile activity of new cardiomyocytes, yields alteration in fibroblasts arrangement. Beside stress distribution, the chemical signals, such as growth factors, proteoglycans and matricellular proteins, mediate the tissue remodeling after injury. Thus, mechanical stimulation together with the complex interconnections among cardiac cells, noncardiac cells and the ECM regulate the cell proliferation and architectural dynamics of the post-infarct.

Here, we identified two useful labels, anti-VIM antibody for post-infarct fibroblasts, and anti-TNC antibody for the de-adhesive ECM protein. Our study demonstrated the localization of both markers around the injury site, predominantly along its outer wall. Vimentin (VIM), an intermediate filament protein, is characteristic for cells under mechanical stress, and it may support the damaged ventricular wall [32]. The most intriguing point is TNC localization at the border zone between the intact myocardium and the injury site. Tenascin-C possesses counter-adhesive properties, and it may loosen cardiomyocytes from the matrix to facilitate their invasion [37,42]. This hypothesis is consistent with in vitro studies demonstrated that TNC loosens the attachment of cardiomyocytes to the substratum, promoting their migration [43]. These data strongly suggest that TNC is a modulator of cardiomyocyte attachments and it contributes to shifting of new myocardium along the supporting fibroblast scaffold. In rodents, TNC is transiently upregulated during the early phase of healing at the interface between infarcted and viable myocardium [43]. However, its expression virtually disappears in the mature infarct. Examination of TNC knockout mice demonstrated defects in recruitment of cardiac myofibroblasts after myocardial injury, indicating its role in remodeling of the infarct tissue [44]. Ectopic delivery of Tenascin-C during the later phase of healing might be one of the promising therapeutic approaches in the treatment of myocardial infarction in humans. Further research focusing on environmental factors influencing the interaction between cardiac and non-cardiac cells will be beneficial towards designing novel therapeutic strategies for improved regeneration of the infarcted mammalian heart.

## Abbreviations

dpci: days post cryoinjury; dps: days post sham; AFOG: Acid Fuchsin Orange-G; H&E: Hematoxylin and Eosin; ECG: electrocardiogram; ECM: extracellular matrix; TPM: Tropomyosin; MEF-2: Myocyte Enhancer Factor-2; VIM: Vimentin; TNC: Tenascin-C; DAPI: 4',6-diamidino-2-phenylindole; TUNEL: Terminal deoxynucleotidyl transferase dUTP nick end labeling;

## Authors' contributions

FC collected all the data, interpreted the experiments, and drafted the manuscript. JV and GR participated in setting up the ECG experiment. AJ designed the study, helped in the data interpretation and wrote the manuscript. All authors read and approved the final manuscript.

## Supplementary Material

Additional file 1 **Three-dimensional-reconstruction of the zebrafish heart from a consecutive series of AFOG-stained sections at 7 dpci**. Several anatomical structures can be seen: the atrium (spongy appearance, light orange), the ventricle (dense muscle tissue, intense orange), the bulbus arteriosus with abundent collagen (dark blue), the atrioventricular valve (green) and the outflow tract between the ventricle and the bulbus arteriosus (green). The post-infarct zone extends from the apex of ventricle to the half-length of the ventricle wall. The outer border of the injured area is surrounded by fibrin (red), and fibrotic tissue. The middle part of the injured area contains a loose collagen network (light blue/green). Click here for file

Additional file 2 **H&E histological analysis of the scar and of the inflammatory response**. **(A-C) **Heart cross-sections stained with Hematoxylin (dark purple) to visualize nuclei and Eosin (pink) to detect proteins. **(A', B' and C') **Higher magnifications of framed area shown in left panels. **(A-A') **At 4 dps, the intact ventricle is surrounded by the compact myocardium. The middle part of the ventricle consists of trabecular myocardium. **(B-B') **At 7 dpci, the scar tissue (dashed line) is infiltrated by inflammatory cells. A network of fibroblasts surrounds the outer border of the infarct. A layer of acellular matrix accumulates at the inner side of this border. **(C-C') **At 14 dpci, the compact myocardium starts to invade the outer margin of the scar. The interior of the post-infarct is composed of a network of spindle-shaped fibroblasts, which is infiltrated by blood cells. cm, compact myocardium; tm, trabecular myocardium, bc, blood cells; f, fibrin; wm, wound margin; fn, fibroblast network in the interior of the scar. Scale bars in **(A-A') **represent 300 μm. Click here for file

Additional file 3 **A new myocardium surrounds the post-infarct area during heart regeneration**. **(A and E) **AFOG staining of a consecutive series of transverse sections of a heart at 14 dpci **(A) **and at 30 dpci **(E) **from the top of the ventricle (left upper corner) to the ventricular apex (right bottom corner); v, ventricle; va, valve; ba, bulbus arteriosus; a, atrium. **(B-D) **Higher magnification of selected images shown in **(A)**. The post-infarct zone (dashed line) containing fibrin (red) and collagen (blue) expands from the apex to approximately a half-length of the ventricular wall. New cardiac tissue (orange) begins to surround the post-infarct (arrows). **(F-H) **Higher magnification of selected images shown in **(E)**. The post-infarct zone (dashed line) is detected only at the level of the artioventricular valves **(F)**, and it is completely replaced by a new myocardium in the apex and the lower part of the ventricle **(G and H)**. **(F) **A wall of cardiac tissue surrounds the remaining collagenous scar (arrows). Click here for file

Additional file 4 **Sham surgery triggers a cell-cycle entry of the cardiomyocytes in the vicinity the epicardium**. **(A-B) **The nuclei of cardiomyocytes express DsRed2-Nuc protein under the control of *cmlc-2 *promoter. Tropomyosin (blue) labels the myocardium, MCM5 (green) is expressed in the mitotic cells, DAPI marks all the nuclei. Proliferating cardiomyocytes are identified by the co-expression of DsRed2-Nuc and MCM5 (circles). **(A' and B') **Higher magnifications of framed area shown in left panels. **(A-A') **Uninjured animals display a very few proliferating cardiomyocytes in the ventricle. **(B-B') **The ventricle of animals at 4 days sham-operation contains an enhanced number of DsRed2-Nuc/MCM5-positive nuclei at the myocardial periphery, underneath the epicardium. Bars in **(A-A') **represent 300 μm. Click here for file

Additional file 5 **Distribution of the endothelial cells in the ventricle at 14 dpci**. **(A) **The endothelial cells express GFP under the control of *tie-2 *promoter [45]. Tropomyosin (blue) labels the myocardium, TNC (red) is expressed in the post-infarct zone. **(A') **Higher magnification of the framed area in **(A) **demonstrates formation of new blood vessels in the post-infarct area. Tenascin-C outlines the boundary between the invading myocardium and the injury site. Scale bar in **(A) **represents 300 μm. Click here for file

Additional file 6 **Fibroblasts with contractile filaments constitute the wall of the post-infarct**. **(A) **Heart section immunostained for a cardiac marker Tropomyosin (red), a myofibroblast marker alpha smooth muscle actin (green) and DAPI (blue). The outer rim of the post-infarct is surrounded by myofibroblasts. **(A') **Higher magnification of the framed area in **(A) **reveals fibroblast-cardiomyocyte coupling at the edge of the invading myocardium (arrowheads). Scale bar in **(A) **represents 300 μm. Click here for file
