# Supplementary figures and images for: The zebrafish heart regenerates after cryoinjury-induced myocardial infarction
Source: BMC Dev Biol. 2011 Apr 7;11:21. doi: 10.1186/1471-213X-11-21 (PMC3078894; doi:10.1186/1471-213X-11-21)

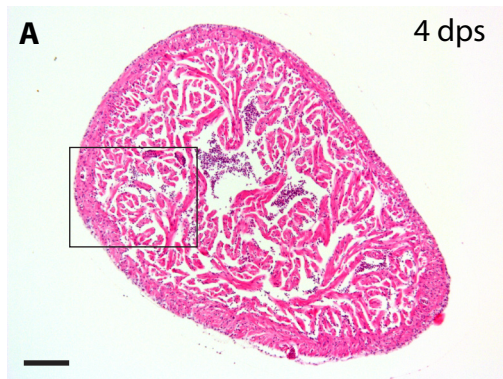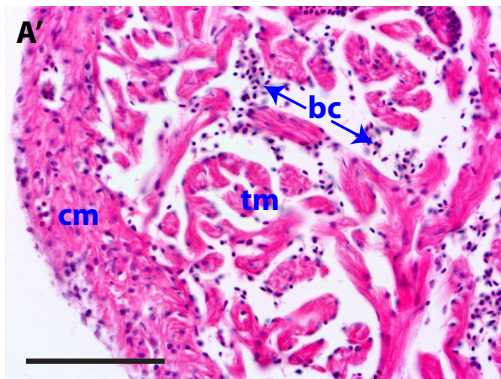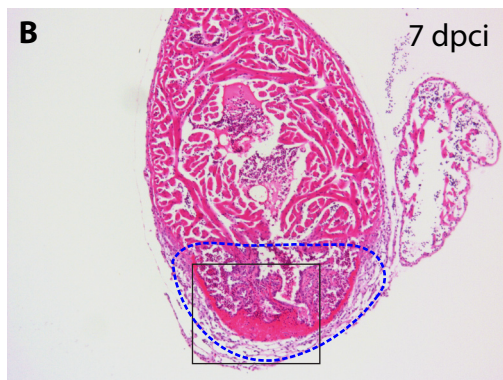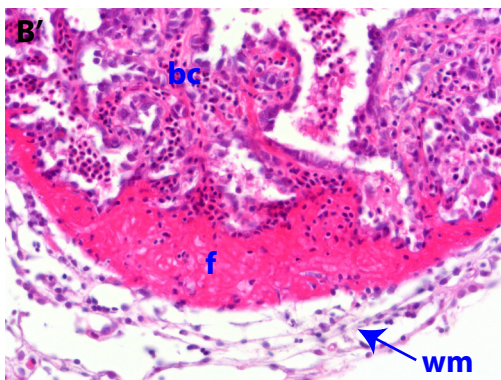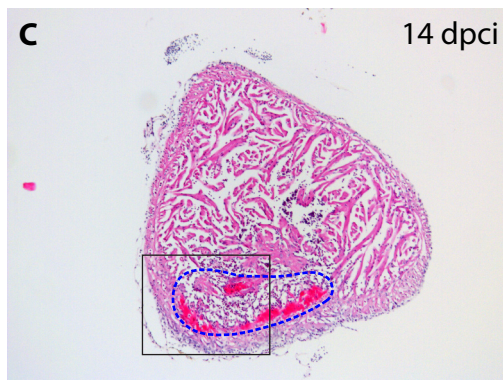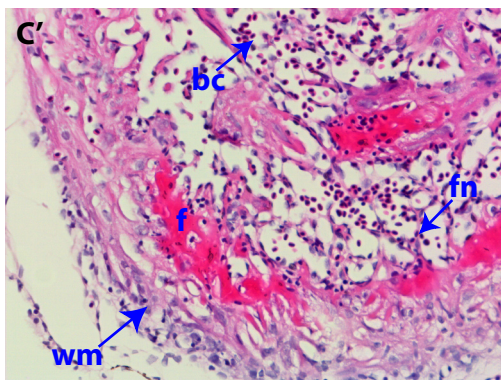

Supplement: Additional file 2 — H&E histological analysis of the scar and of the inflammatory response. (A-C) Heart cross-sections stained with Hematoxylin (dark purple) to visualize nuclei and Eosin (pink) to detect proteins. (A', B' and C') Higher magnifications of framed area shown in left panels. (A-A') At 4 dps, the intact ventricle is surrounded by the compact myocardium. The middle part of the ventricle consists of trabecular myocardium. (B-B') At 7 dpci, the scar tissue (dashed line) is infiltrated by inflammatory cells. A network of fibroblasts surrounds the outer border of the infarct. A layer of acellular matrix accumulates at the inner side of this border. (C-C') At 14 dpci, the compact myocardium starts to invade the outer margin of the scar. The interior of the post-infarct is composed of a network of spindle-shaped fibroblasts, which is infiltrated by blood cells. cm, compact myocardium; tm, trabecular myocardium, bc, blood cells; f, fibrin; wm, wound margin; fn, fibroblast network in the interior of the scar. Scale bars in (A-A') represent 300 μm. [file 1471-213X-11-21-S2.PDF]

**A** 14 dpci - Intact muscle Fibrin Collagen

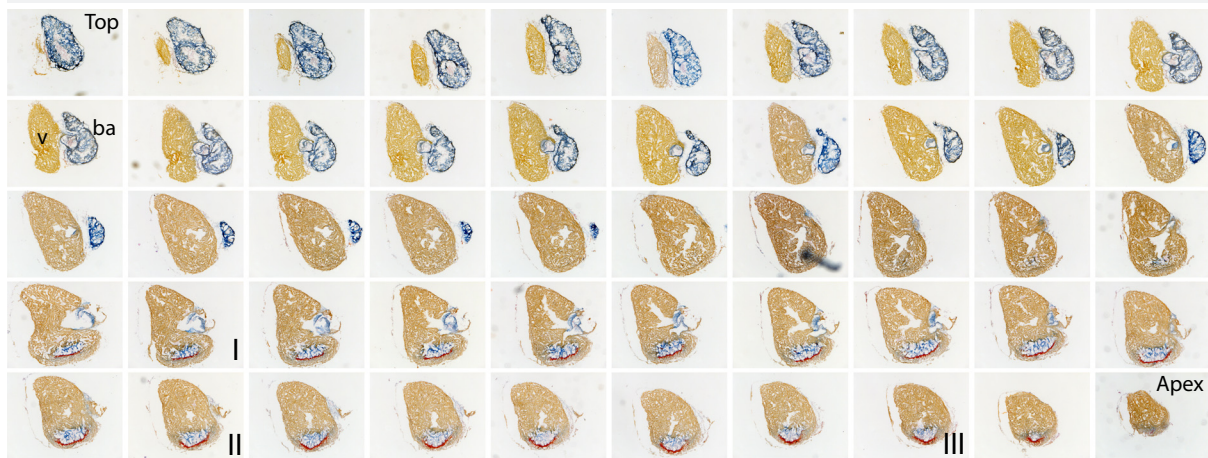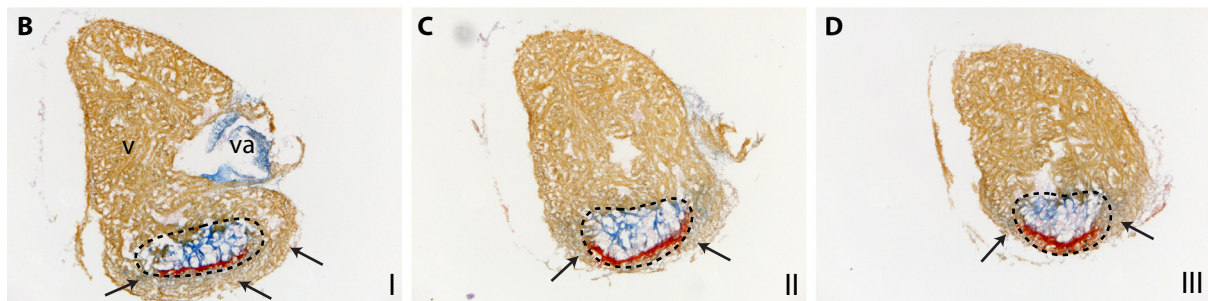

**E** 30 dpci - Intact muscle Fibrin Collagen

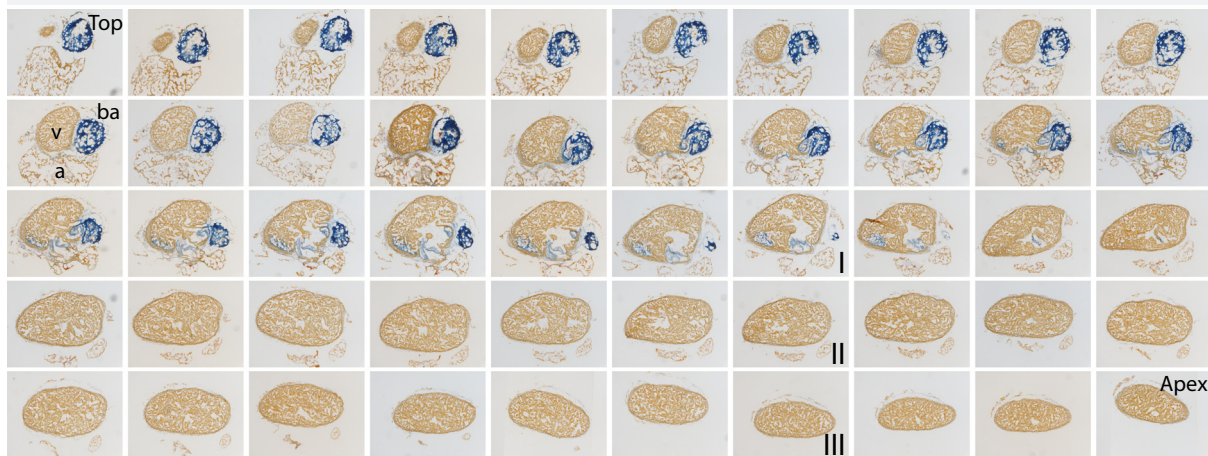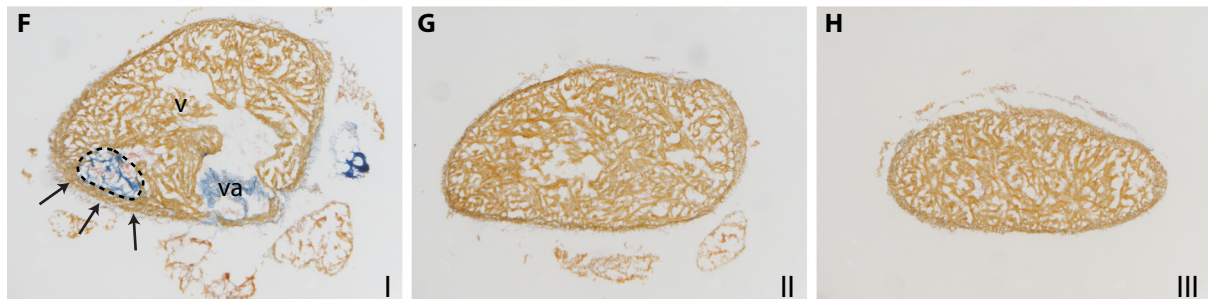

Supplement: Additional file 3 — A new myocardium surrounds the post-infarct area during heart regeneration. (A and E) AFOG staining of a consecutive series of transverse sections of a heart at 14 dpci (A) and at 30 dpci (E) from the top of the ventricle (left upper corner) to the ventricular apex (right bottom corner); v, ventricle; va, valve; ba, bulbus arteriosus; a, atrium. (B-D) Higher magnification of selected images shown in (A). The post-infarct zone (dashed line) containing fibrin (red) and collagen (blue) expands from the apex to approximately a half-length of the ventricular wall. New cardiac tissue (orange) begins to surround the post-infarct (arrows). (F-H) Higher magnification of selected images shown in (E). The post-infarct zone (dashed line) is detected only at the level of the artioventricular valves (F), and it is completely replaced by a new myocardium in the apex and the lower part of the ventricle (G and H). (F) A wall of cardiac tissue surrounds the remaining collagenous scar (arrows). [file 1471-213X-11-21-S3.PDF]

DAPI TPM

*cmlc::DsRed2-Nuc* MCM5

*cmlc::DsRed2-Nuc* MCM5

A

Uncut

A'

B

4 dps

B'

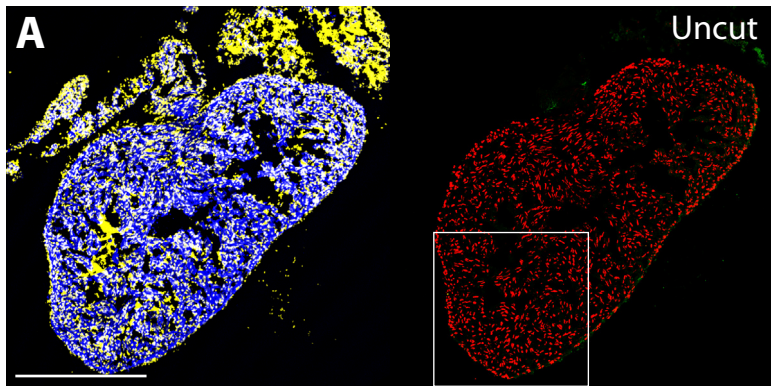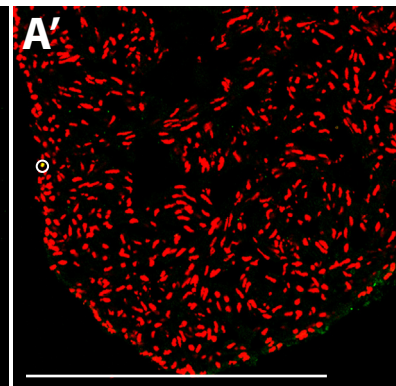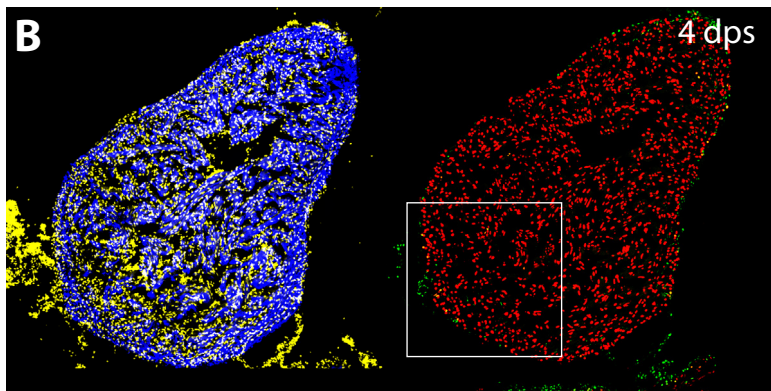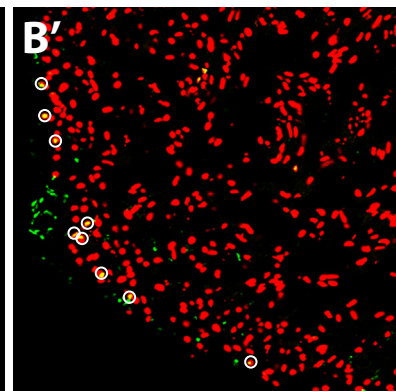

Supplement: Additional file 4 — Sham surgery triggers a cell-cycle entry of the cardiomyocytes in the vicinity the epicardium. (A-B) The nuclei of cardiomyocytes express DsRed2-Nuc protein under the control of cmlc-2 promoter. Tropomyosin (blue) labels the myocardium, MCM5 (green) is expressed in the mitotic cells, DAPI marks all the nuclei. Proliferating cardiomyocytes are identified by the co-expression of DsRed2-Nuc and MCM5 (circles). (A' and B') Higher magnifications of framed area shown in left panels. (A-A') Uninjured animals display a very few proliferating cardiomyocytes in the ventricle. (B-B') The ventricle of animals at 4 days sham-operation contains an enhanced number of DsRed2-Nuc/MCM5-positive nuclei at the myocardial periphery, underneath the epicardium. Bars in (A-A') represent 300 μm. [file 1471-213X-11-21-S4.PDF]

TPM TNC tie2::GFP

A

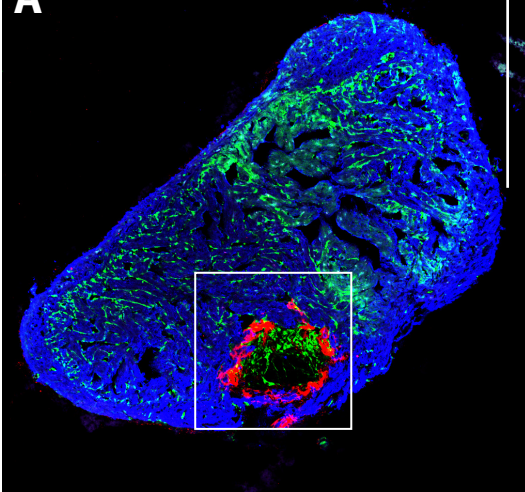

TNC tie2::GFP

14 dpf

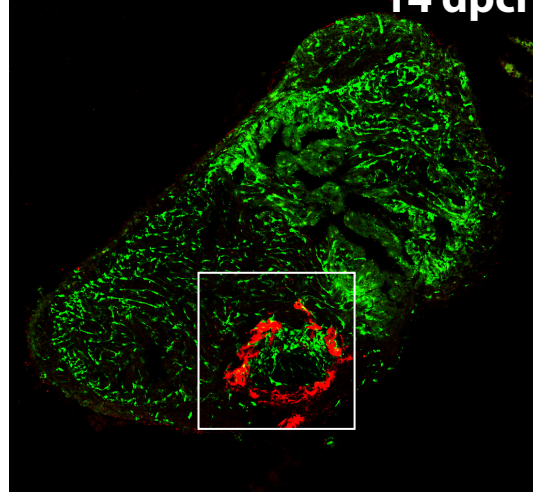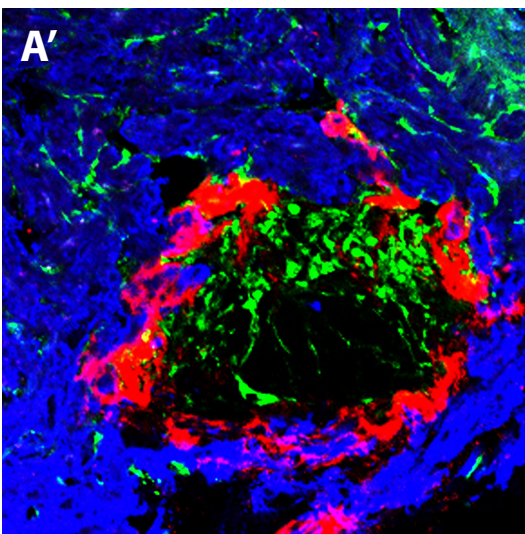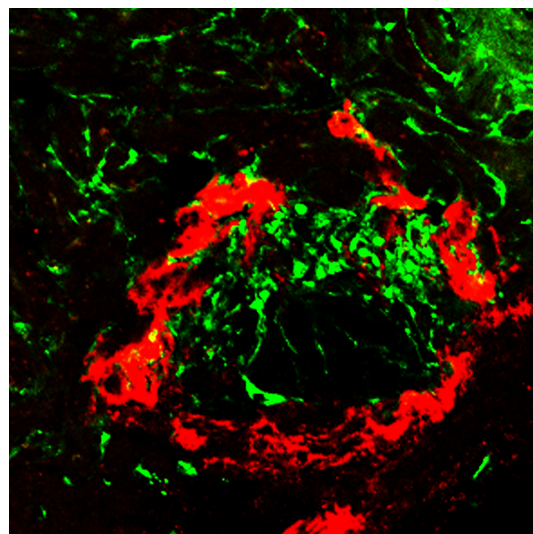

Supplement: Additional file 5 — Distribution of the endothelial cells in the ventricle at 14 dpci. (A) The endothelial cells express GFP under the control of tie-2 promoter [45]. Tropomyosin (blue) labels the myocardium, TNC (red) is expressed in the post-infarct zone. (A') Higher magnification of the framed area in (A) demonstrates formation of new blood vessels in the post-infarct area. Tenascin-C outlines the boundary between the invading myocardium and the injury site. Scale bar in (A) represents 300 μm. [file 1471-213X-11-21-S5.PDF]

DAPI TPM  $\alpha$ -SMA

TPM  $\alpha$ -SMA

A

7 dpci

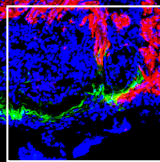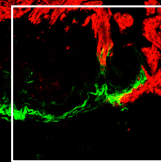

A'

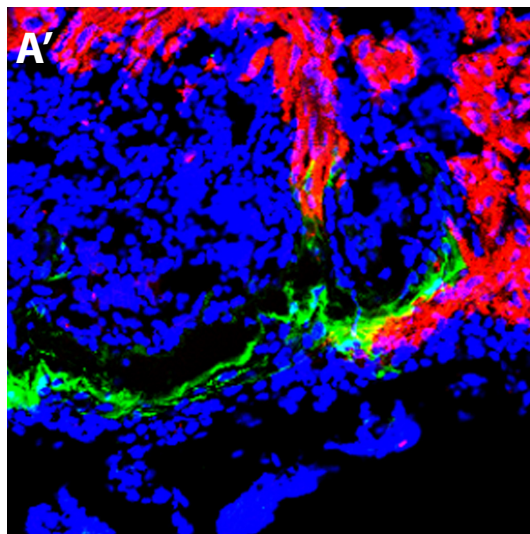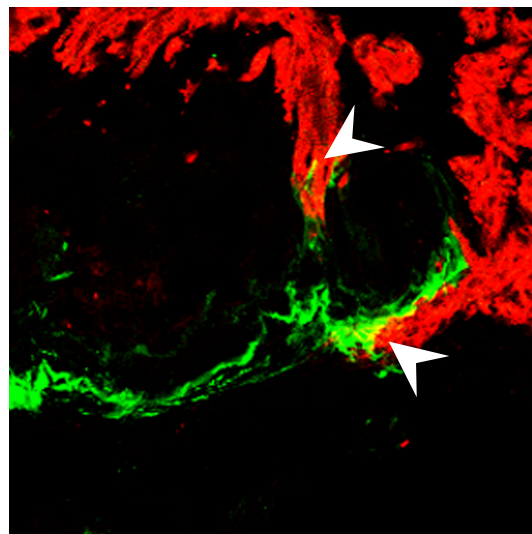

Supplement: Additional file 6 — Fibroblasts with contractile filaments constitute the wall of the post-infarct. (A) Heart section immunostained for a cardiac marker Tropomyosin (red), a myofibroblast marker alpha smooth muscle actin (green) and DAPI (blue). The outer rim of the post-infarct is surrounded by myofibroblasts. (A') Higher magnification of the framed area in (A) reveals fibroblast-cardiomyocyte coupling at the edge of the invading myocardium (arrowheads). Scale bar in (A) represents 300 μm. [file 1471-213X-11-21-S6.PDF]
